# Clinical Utility of ctDNA Analysis in Lung Cancer—A Review

**DOI:** 10.3390/arm93030017

**Published:** 2025-06-12

**Authors:** Kamil Makar, Agata Wróbel, Adam Antczak, Damian Tworek

**Affiliations:** 1 Department of General and Oncological Pulmonology, Medical University of Lodz, Stefana Zeromskiego 113, 90-549 Lodz, Poland; kamil.makar@umed.lodz.pl (K.M.); adam.antczak@umed.lodz.pl (A.A.); damian.tworek@umed.lodz.pl (D.T.); 2 Faculty of Medicine, Medical University of Lodz, Pomorska 251, 92-213 Lodz, Poland

**Keywords:** ctDNA, cfDNA, liquid biopsy, lung cancer, NSCLC, cancer diagnostics

## Abstract

**Highlights:**

**What are the main findings?**

- ctDNA can be detected using both NGS techniques and various forms of PCR.
- ctDNA reflects changes in the tumor’s genetic makeup.
- Liquid biopsy with ctDNA is clinically approved in genetic profiling in NSCLC.

**What is the implication of the main finding?**

- ctDNA has potential as a biomarker for cancer relapse prediction and treatment efficacy.

**Abstract:**

Circulating free DNA (cfDNA) is genetic material released from various cells into bodily fluids. Among its fractions, circulating tumor DNA (ctDNA) originates from tumor cells and reflects their genetic material, including mutations and epigenetic changes. Methods commonly employed for detecting ctDNA in blood include next-generation sequencing (NGS) and various types of PCR. The presence of ctDNA can be utilized in liquid biopsies for many diagnostic purposes related to various cancers. It is a minimally invasive method of sampling molecular compounds from tumor cells. In this paper, we focus on current knowledge regarding the liquid biopsy of blood ctDNA in the context of lung cancer, one of the leading causes of cancer-related mortality. Currently, as a clinically approved method, liquid biopsy serves as a complementary technique in NSCLC diagnostic and genetic profiling. Other applications of liquid biopsy that are still being investigated include the detection of minimal residual disease (MRD) after curative treatment and response monitoring to systemic treatment. This review discusses current and future potential directions for the development and implementation of ctDNA for patients with NSCLC.

## 1. Introduction

Globally, lung cancer is the most commonly diagnosed cancer and a leading cause of cancer-related death in both genders [1,2]. The tumor is most frequently detected in advanced stages, resulting in unsatisfactory treatment outcomes. Histologically, lung cancer can be divided into two main categories: small-cell lung cancer (SCLC) and non-small-cell lung cancer (NSCLC). SCLC constitutes approximately 15% of all cases, while NSCLC is responsible for about 85% of the remaining cases and includes different subtypes, such as squamous-cell carcinoma, adenocarcinoma, and large-cell carcinoma [3,4]. For all types of lung cancer, smoking is the primary risk factor, exhibiting the strongest association with small-cell lung cancer and squamous-cell carcinoma. However, approximately 25% of lung cancer cases are not associated with smoking. Among patients who have never smoked, adenocarcinoma is the most frequent subtype [5]. Despite advancements in diagnostic procedures and treatment methods for lung cancer, it still has a poor prognosis, marked by a low 5-year survival rate [6,7,8]. This article discusses the current and potential applications of liquid biopsy using cell-free DNA in the diagnostic and therapeutic management of lung cancer.

**Figure 1:**
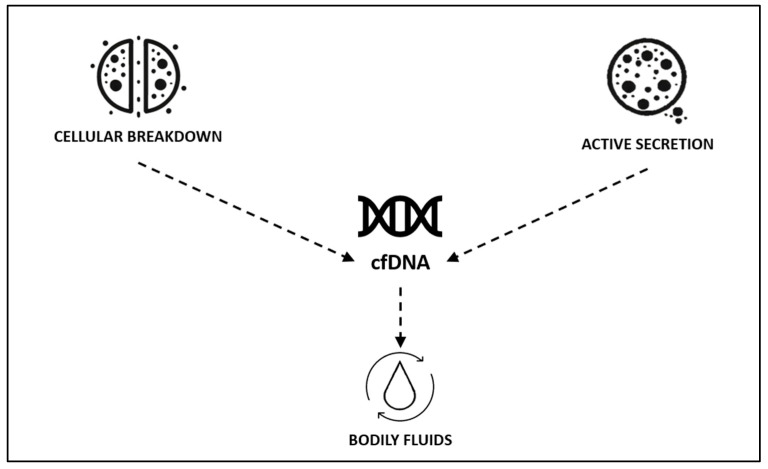
Potential sources of cfDNA in bodily fluids.

Cell-free DNA (cfDNA) is defined as any DNA present in the extracellular environment. cfDNA can be detected in various body fluids, like blood, bile, lymph, urine, saliva, etc., from both healthy and diseased individuals [9,10,11]. The two main sources of cfDNA are cellular breakdown and active secretion. Cell death can occur through apoptosis, necrosis, oncosis, and pyroptosis. Additionally, other DNA release mechanisms include phagocytosis, neutrophil release, excision repair, and the terminal differentiation of erythroid cells (Figure 1). During active secretion, released DNA is encapsulated into vesicles or bound to glycolipoproteins [9,10,12,13].

Circulating tumor DNA (ctDNA) is a subset of cell-free DNA and has potential as a biomarker for enhancing diagnosis, monitoring, and treatment in various types of cancers. ctDNA specifically refers to DNA derived from cells within the tumor compartment, including malignant tumor cells and adjacent cells, such as stromal cells, endothelial cells, and immune cells [9]. Moreover, the primary or metastatic tumor site can be a source of detached circulating tumor cells (CTCs) and released exosomes. CTCs have the ability to enter the circulation through vessel walls. All of these components could be potential sources of ctDNA [14].

The majority of ctDNA is highly fragmented, with an average size of 100–200 base pairs [15]. Higher levels of ctDNA in blood have been observed in cancer patients compared to healthy individuals. Additionally, a well-established correlation between ctDNA concentration and tumor advancement has been demonstrated [16,17,18,19]. This phenomenon can be explained by extensive processes of cell death in large tumors, particularly in advanced stages [20].

The proportion of ctDNA within total cfDNA differs widely, ranging from less than 0.1% in the early stages to over 50% in late-stage cancer with significant tumor burden [18,21,22,23]. Discriminating ctDNA from cfDNA is crucial for molecular tumor analysis. It is feasible because ctDNA originates from malignant cells and contains the same genetic information that is absent in normal cfDNA. The tumor-specific abnormalities of ctDNA, such as point mutations, epigenetic changes, and copy number variations, are inherited from the tumor of origin [24].

## 2. Liquid Biopsy

Tissue biopsy is a standard procedure in oncology for diagnosing cancer and tailoring treatment plans. Biopsy allows for the determination of the histological type of cancer and, due to molecular assays, the characterization of the tumor’s genetic profile. However, it has some serious disadvantages. First of all, tissue biopsy is an invasive procedure, which carries a risk of complications and unsuccessful attempts to collect the sample. Depending on the location of the lesion, obtaining a tissue biopsy may be difficult, and as a result, the amount of material collected may be too small to perform all required molecular tests. These events delay the initiation of targeted treatment. Furthermore, tissue sampling provides only a static and spatially limited genetic profile from the specific fragment removed from the tumor [14,15,25,26]. Cancers change over time, resulting in genetic heterogeneity within the tumor and between the primary and metastatic sites. Tumor heterogeneity hinders the choice of the initial therapeutic approach and makes it difficult to adjust treatment in the case of acquired resistance [14,27]. Therefore, a single biopsy might miss crucial genetic drivers. However, repeating the biopsy involves exposing the patient to complications. Consequently, liquid biopsy, as a non-invasive approach that analyzes ctDNA from blood samples, has gained acceptance as a valuable tool in clinical practice. During this procedure, we can isolate not only ctDNA, which we focus on in this paper, but also other particles such as CTCS, circulating RNA, and exosomes, which can also provide new diagnostic possibilities [28].

Liquid biopsy provides additional insights into the genetic alterations of cancer. This approach contributes to a comprehensive understanding of the constant changes in the tumor’s genetic makeup, thereby aiding in making well-informed decisions about treatment. Liquid biopsy has been extensively investigated in recent studies. It serves as a non-invasive substitute for tumor assessment and holds potential for numerous clinical applications [29]. Despite many advantages, liquid biopsy cannot entirely replace standard procedures. In an article by Adashek J. et al., the authors depict both the advantages and disadvantages of liquid biopsy, noting that while it detects many mutations, genes, etc., we still need accurate identification of the tumor location, which is possible through traditional biopsy [30].

Although ctDNA can be found in various body fluids, the material for liquid biopsy is typically obtained from peripheral blood due to easy access and the abundance of molecular information [15,31,32]. Due to the very small amounts of ctDNA diluted within a large volume of blood, laboratory analysis is challenging and requires careful consideration of various pre-analytical factors affecting the quality of the collected material and the sensitivity of the ctDNA assay results. The most important variables are collected blood volume, interval time from blood collection to material isolation, centrifugation protocol, kits used for further analysis, and storage temperature—all of which can impact the assay quality [33,34,35,36,37]. Plasma is preferred over serum due to a lower risk of contamination by genomic DNA released from the clotting process [38,39]. If further analyses are needed, the long-term storage temperature of centrifuged samples should be at least −80 °C [39].

## 3. Methods of ctDNA Detection

The analysis of ctDNA requires highly sensitive and specific techniques such as next-generation sequencing (NGS) or various types of PCR. The majority of commercial kits are based on real-time PCR, which detects particular mutations and requires designed primers that bind to defined sites in DNA. NGS holds an advantage over PCR in its ability to detect a significantly broader range of abnormalities by comparing entire genes or even the whole genome to the reference. It can also identify previously unknown genetic changes across broad regions in a non-targeted manner [31,40,41,42,43].

Differentiating ctDNA from cfDNA is feasible due to specific genomic alterations present in ctDNA. These alterations, including point mutations, gene amplifications, deletions, translocations, or epigenetic changes, are characteristic of the cancer cell genome [28,29,32,44,45,46]. The tumor burden can be assessed by measuring variant allele frequency (VAF), a metric used in genetics to quantify the fraction of DNA sequencing reads that contain a specific genetic variant (mutation) within a genetic locus. The higher the VAF, the larger the proportion of DNA in the sample that carries the mutation. For example, if the DNA sequencing of a specific genomic region results in 100 reads, and 40 of those contain a mutation while 60 do not, the VAF would be 40% [47]. A positive correlation between tumor volume and ctDNA VAF has been demonstrated in NSCLC [48]. However, VAF is only an indirect indicator of tumor size, as tumors are highly heterogeneous and consist of various cell types, not all of which carry the same mutation or any mutation at all. Additionally, tumor location and cellular activity play a significant role. A relatively small tumor with high cellular activity can release large amounts of DNA despite its small size. Another detectable feature of ctDNA is the presence of epigenetic changes, such as aberrant DNA methylation patterns. Methylation alterations are highly specific to tumor cells and can serve as cancer biomarkers, especially in the absence of detectable mutations in ctDNA [49,50,51,52,53].

ctDNA detected in blood represents the real-time status of the tumor genome and contains the same genetic alterations as the solid tumor from which it originates. It offers the potential for non-invasive sampling, which enables tracking tumor evolution over time. Moreover, blood sampling for ctDNA can detect mutations from multiple regions and may be more representative than traditional tissue biopsy, which reflects only a part of a tumor. This approach can partially address the challenges of tumor heterogeneity and the limited availability of traditional biopsy samples [23,54,55,56,57,58].

## 4. Lung Cancer Screening and Its Early Detection

It is extremely rare to diagnose lung cancer at an early stage of the disease, and most such diagnoses are incidental. This is mainly due to the absence of symptoms for a long time during the course of the disease. Of all screening methods studied so far, only low-dose computed tomography (LDCT) has been proven to reduce mortality in high-risk groups [59,60]. However, it also has its drawbacks. Patients who undergo regular LDCT screening often receive false positive results, which lead to confusion and the need for excessive invasive diagnostic procedures, exposing patients to complications [61]. Moreover, each LDCT scan is associated with exposure to ionizing radiation. Therefore, there is no effective method for detecting lung cancer in the general population, despite it being one of the most common and poor-prognosis cancers. That is why there is a need for a complementary diagnostic tool, enabling the detection of early lung cancer and differentiation between cancer and benign changes.

There is already some evidence that ctDNA can fulfill this role. Liang W. et al. developed a diagnostic prediction model, based on methylation pattern differences between malignant and benign tumors. The model was able to detect lung cancer in stages Ia and Ib and differentiate between malignant and benign changes with high sensitivity [62]. A different perspective is presented in a study by Bittla et al., where the authors argue that ctDNA would be much more effective in monitoring the disease and predicting its course rather than in screening. The reviewed literature showed no significant utility of ctDNA in detecting tumors smaller than 1 cm in diameter. Despite such conclusions, the authors do not dismiss ctDNA as a cancer screening method. They emphasize that further studies are needed to determine its utility in the early detection of lung and other cancers. It is important to note that ctDNA should be regarded as an adjunctive method that cannot replace currently used and standardized diagnostic techniques, because even if an assay detects a mutation, it cannot localize the disease [63].

## 5. The Utilization of ctDNA in Routine Clinical Practice

ctDNA has already been incorporated into daily practice for the management of some types of cancers, such as breast, colorectal, or gastric cancers and NSCLC. In many others, such as ovarian, pancreatic cancer, or melanoma, meta-analyses show that it could be useful tool, but further investigation is needed. The use of ctDNA is similar across various cancer types and focuses primarily on diagnostics, particularly identifying mutations, which allows clinicians to personalize therapy based on the molecular profile of the cancer, select appropriate treatments, and detect early signs of recurrence or progression [64,65,66,67,68,69].

**Figure 2:**
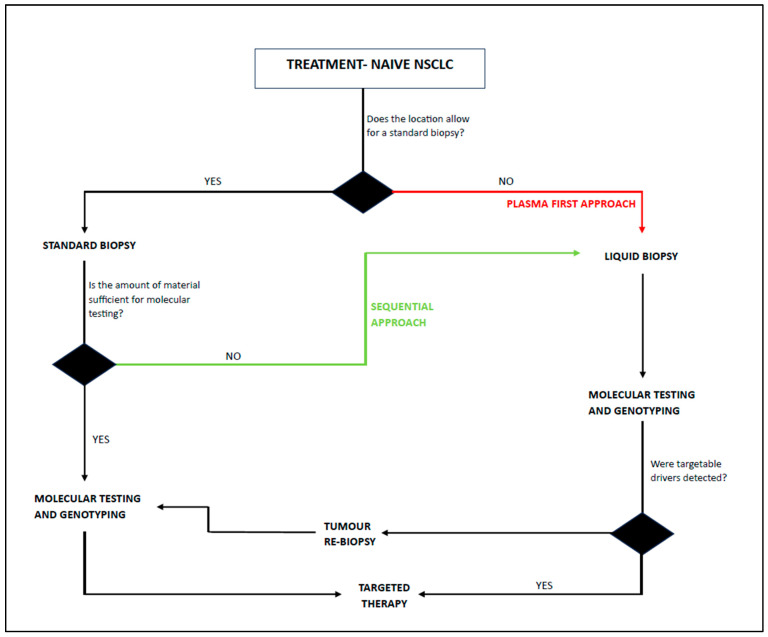
Proposed diagnostic algorithm for ctDNA application for tumor genotyping in advanced NSCLC patients. NSCLC—non-small-cell lung cancer.

In the case of NSCLC, liquid biopsy has been incorporated into routine management in certain clinical situations. For treatment-naive advanced NSCLC patients with an unknown genotype, ctDNA can be used in different approaches. When the location of a tissue biopsy is challenging and the obtained material is insufficient for molecular testing, a “plasma first” strategy can be applied. In the absence of targetable drivers in plasma, a rebiopsy should be considered. When tissue is available for genotyping, but the quantity of material is uncertain, ctDNA can be analyzed concurrently as a “complementary approach” or “sequentially” in the case of incomplete tumor genotyping. Eventually, a “plasma first” approach can be implemented in cases of acquired resistance during tyrosine kinase inhibitor (TKI) therapy for the identification of resistance mechanisms with a repeat tissue biopsy if ctDNA is not informative (Figure 2) [70,71,72].

Interesting findings were demonstrated in the LIBELULE study where patients with radiological suspicion of lung cancer were divided into two groups to evaluate the time to treatment initiation. In the control group, patients underwent standard diagnostic procedures, while in the experimental group, liquid biopsy was additionally performed. The study found that the use of liquid biopsy shortened the time required for genomic analysis and accelerated treatment initiation in patients eligible for first-line systemic treatment, particularly those with advanced non-squamous NSCLC and actionable mutations. Furthermore, liquid biopsy increased the detection rate of targetable genomic alterations [73].

A major clinical limitation for ctDNA is its sensitivity, with a risk of false negative results, due to many variables such as a low level of plasma DNA, assay imperfections, or non-shedding tumors when disease burden is low. Therefore, ctDNA analysis should be performed using standardized and clinically validated procedures for sample collection and processing. Given the large number of potential target alterations, the preferred method for DNA analysis is NGS rather than single-gene PCR approaches [74,75,76].

## 6. Applications of ctDNA in Lung Cancer

### 6.1. Treatment with Curative Intention

Even in the early, resectable stages of lung cancer, a high risk of disease recurrence remains, sometimes many years after treatment [77,78]. In the postoperative period, active monitoring is crucial to detect residual disease or identify recurrence early during the follow-up. Currently, monitoring relies on repeated radiological examinations, which are limited by resolution and can only detect macroscopic changes. To improve treatment outcomes, there is a need for a more accurate tool that could identify a high likelihood of residual or recurrent cancer before changes become visible on chest CT scans. In a study conducted on 330 cases of operable lung cancer in stages I-III, ctDNA was measured before surgery, 3 days after surgery, and one month after surgery. For ctDNA measurement, an NGS panel of 769 cancer-related genes was used. Of the ctDNA-positive patients before surgery, 46.4% (32 out of 69) experienced disease recurrence. Among ctDNA-negative patients, this rate was significantly lower at 14.6% (38 out of 261). Furthermore, patients with detectable preoperative ctDNA had a significantly shorter recurrence-free survival compared to ctDNA-negative patients. The utility of ctDNA in detecting residual disease was then examined. Patients with detectable ctDNA at 3 days and/or one month postoperatively were defined as MRD-positive, while those with no detectable ctDNA at any postoperative time point were classified as MRD-negative. During follow-up, MRD-positive patients had a higher recurrence rate (80.8%, 21 out of 26 patients) compared to MRD-negative patients (16.2%, 49 out of 303 patients). Both preoperative and postoperative ctDNA detection were independent risk factors for disease recurrence. MRD-positive status was a stronger predictor of recurrence than other clinical and pathological variables, such as age, comorbidities, tumor size, histopathological subtype, and TNM stage. Among MRD-positive patients, the addition of adjuvant therapy improved recurrence-free survival; however, surprisingly, in the MRD-negative group, the addition of adjuvant therapy worsened this outcome [79].

Similar findings emerged from another study, where all MRD-positive patients at any time point after surgery exhibited significantly lower progression-free survival and long-term survival rates. Furthermore, at the 36-month time point from MRD, the progression-free rate was 0% for the MRD-positive group and 93% for the MRD-negative group, highlighting the very high specificity of ctDNA in predicting disease recurrence. The detection of ctDNA preceded disease progression, as defined by RECIST 1.1 criteria on CT scans, in 72% of cases, with a median lead time of approximately 5 months [80]. The ctDNA analysis served as a reliable indicator in cases of negative or equivocal CT imaging, where distinguishing between tumor recurrence and treatment-related changes or other processes was not possible through radiological means [81]. The referenced meta-analyses, including 1686 [65] and 1251 [82] patients, respectively, from a total of 31 articles, confirm that positive ctDNA status before surgery, after surgery, and during long-term postoperative monitoring is an independent risk factor for disease recurrence. The specificity of ctDNA MRD in predicting recurrence is highly sensitive (86–95%) but moderately specific (41–76%).

Currently, patients at stages II-IIIA are candidates for adjuvant chemotherapy, which modestly improves the five-year survival rate by approximately 4–5% [83,84,85,86]. The detection of ctDNA in the blood after lung resection can identify patients with minimal residual disease, who may particularly benefit from adjuvant therapy [87]. This approach allows for personalized treatment and reduces the risk of adverse effects from unnecessary systemic treatment.

Perioperative treatment is a beneficial option for patients with a resectable tumor. Two phase 3 clinical trials, called CheckMate 77T and AEGEAN, delivered similar findings, demonstrating significant benefits from the addition of immunotherapy to neoadjuvant chemotherapy, followed by continued immunotherapy after radical surgery in patients with stage II-IIIB disease [88,89]. Treatment arms incorporating immunotherapy in addition to chemotherapy showed a higher rate of ctDNA clearance prior to surgery, which correlated with higher pathological complete response rates (pCR, defined as the absence of viable cancer cells in post-surgery material) and improved event-free survival (EFS) post-surgery. Moreover, ctDNA clearance identifies patients likely to experience longer EFS earlier and more sensitively than pCR [88,89]. ctDNA, as a more dynamic marker, can be evaluated during neoadjuvant treatment, providing an advantage in early response predictions.

### 6.2. Treatment Monitoring

Decisions about cancer treatment are guided by tumor dynamics observed in serial chest CT scans. Control CT scans involve radiation exposure; therefore, they are repeated at specific intervals, which can delay therapeutic adjustment when treatment is ineffective. Moreover, the interpretation is not always straightforward due to various treatment-related changes in the lungs, such as post-radiation inflammation, immune-related inflammation, or pseudoprogression [90].

Many studies have investigated the use of serial quantitative ctDNA assessments as a real-time molecular biomarker for monitoring treatment efficacy and predicting clinical outcomes. Wang et al. summarize the predictive role of ctDNA in advance or metastatic non-resectable NSCLC patients treated with immunotherapy. In this review based on 1017 patients from 10 studies, it was well-proven that early reduction in ctDNA concentration after the initiation of treatment was associated with improved PFS and OS [91]. In most studies, a decrease in ctDNA is evaluated within four weeks of checkpoint inhibitor treatment [92,93,94]. The analysis of ctDNA concentration dynamics typically identifies three patterns: (1) complete clearance (molecular responders), (2) initial decline followed by increase (responders with acquired resistance), and (3) continuous rise (non-responders) [92]. The cut-off for molecular response was proposed as a >50% reduction in variant allele fraction from baseline [94,95,96]. In Thompson et al.’s study, the median PFS was 14.1 months for responders and 4.4 months for non-responders, while the median OS was 22.1 months for responders and 12 months for non-responders. Interestingly, no association was found between PDL-1 status and molecular response to pembrolizumab [96]. The longitudinal assessment of ctDNA led to the conclusion that the clearance of ctDNA at any time point during the treatment course was an independent predictive factor for longer PFS and OS, regardless of treatment type [97]. Data from a study of SCLC patients treated with chemotherapy showed that a VAF increase provided evidence of disease progression before conventional imaging [98].

In a study by Anagnostou et al., a high concordance between ctDNA and radiological Response Evaluation Criteria in Solid Tumors (RECIST) was observed, with a sensitivity of 82% and a specificity of 75%. The median time to ctDNA response was 2.1 months. Consistent with findings from other studies, patients demonstrating a molecular response exhibited longer PFS and OS [99].

The most significant studies evaluating ctDNA dynamics in relation to targeted therapy have been conducted in patients treated with EGFR inhibitors. A meta-analysis by Phan et al. indicates that prior-treatment EGFR mutation positivity in both tumor tissue and plasma, as well as post-treatment persistence or recurrence of EGFR mutations, is a worse survival prognostic factor [100]. Moreover, the detection of such mutations by liquid biopsy can precede radiologic progression by months [101,102]. On the other hand, similar to findings in immunotherapy, the rapid clearance of ctDNA carrying sensitizing EGFR mutations following treatment initiation with osimertinib was associated with improved outcomes [103]. A fact worth noting is that a decrease in ctDNA concentration can help in discriminating between progression and pseudoprogression during the course of treatment [104,105].

The most important research studies on the use of ctDNA in lung cancer monitoring are summarized in Table 1.

## 7. Discussion

Liquid biopsy, a molecular technique based on ctDNA analysis, holds significant promise for advancing personalized treatment in patients with NSCLC. A fundamental aspect of liquid biopsy is the clear distinction between ctDNA and cfDNA. cfDNA comprises DNA fragments released into the bloodstream from both normal and tumor cells, while ctDNA is a tumor-derived fraction of cfDNA that harbors cancer-specific genetic alterations. 

ctDNA detection typically relies on the identification of tumor-specific mutations using NGS panels. Cases where a tumor lacks detectable mutations or harbors rare alterations are more challenging. Implementing broad NGS panels at baseline can increase the likelihood of identifying mutations and facilitate subsequent ctDNA-based monitoring, particularly for assessing minimal residual disease (MRD) and the early detection of recurrence. This distinction is critical for understanding ctDNA’s limitations and optimizing its clinical application in lung cancer management.

Currently, liquid biopsy is clinically approved for detecting actionable mutations in treatment-naive NSCLC patients and in cases of acquired resistance to first- or second-generation tyrosine kinase inhibitor (TKI) therapy. Several studies have demonstrated that complementary concurrent plasma and tissue NGS genotyping increases the detection rate of therapeutically targetable mutations by up to 15% [107,108,109]. Moreover, ctDNA-based genotyping is non-inferior to tissue biopsy and significantly reduces the time to treatment initiation due to shorter turnaround times [107,110,111]. 

However, detecting certain aberrations, such as ALK and ROS1 rearrangements using ctDNA, is limited by the wide spectrum of fusion partners, requiring highly specific NGS assays. Exemplary studies involving patients with ALK and ROS1 rearrangements have reported ctDNA-based sensitivities of 54.2% and 50%, respectively [112,113]. These findings highlight the need to refine ctDNA detection methods to enhance their sensitivity and specificity for various genetic alterations in NSCLC. 

Another important limitation of ctDNA is the fact that the detection range is influenced by various clinical and pathological factors. The fraction of ctDNA varies greatly depending on tumor stage and burden. An advanced stage with a high tumor burden is more likely to shed ctDNA due to extensive apoptosis or necrosis [18,114,115,116]. Moreover, the TRACERx study found that a non-adenocarcinoma histological type, lymphovascular invasion, and a high Ki-67 proliferation index are independent predictors for ctDNA positivity [48].

Studies analyzing the sensitivity and specificity of liquid biopsy consistently report high specificity, often exceeding 95% and reaching up to 100%, regardless of disease stage [117]. However, sensitivity is highly variable and notably lower in early-stage disease. A meta-analysis by Franzi et al. on EGFR mutations reports an overall sensitivity of 59%, with sensitivity stratified by stage at 27% for low-stage disease (I-IIIA) and 75% for advanced-stage disease (IIIB-IV) [118]. The high specificity of liquid biopsy, indicating low rates of false positive results, makes it a reliable tool for confirming mutations in NSCLC patients. However, negative results should be interpreted with caution, especially in early-stage disease.

Applications that are not yet clinically approved include the use of ctDNA to detect MRD in patients undergoing surgical tumor resection with or without perioperative therapy, as MRD serves as a strong prognostic indicator for disease recurrence. In advanced or metastatic NSCLC, serial ctDNA level assessments show high concordance with RECIST criteria, enabling earlier prediction of treatment efficacy. The role of ctDNA in lung cancer screening and early detection remains uncertain.

## 8. Conclusions

Although ctDNA is the subject of extensive research, a major issue remains the lack of technological standardization. Many laboratory and clinical variables can influence ctDNA assays, changing their sensitivity and specificity. While ctDNA assays are commonly used in clinical practice for molecular profiling, other applications such as treatment monitoring, MRD detection, or early detection require more data to establish the role of ctDNA as a biomarker.

We hope that further studies based on large patient populations will support the inclusion of liquid biopsy into clinical practice as a tool guiding clinical decisions and improving outcomes in lung cancer management.

## Figures and Tables

**Table 1:** Summary of research conducted between 2020 and 2025 on the potential clinical applications of ctDNA in lung cancer management.

Clinical Application of ctDNA	First Author	Year	Number of Subjects	Results	*p*	Limitations	Strenghts
MRD detection	Xia [79]	2022	330	The presence of postoperative MRD (ctDNA positivity at postoperative period) was a strong predictor for disease relapse.	<0.001	Mixed I-III stage cohort with most patients in stage I.	Big cohort
	Chen [65]	2023	1686	Positive ctDNA status at the baseline, postoperative, or longitudinal timepoints determine higher risk of recurrence. Persistent ctDNA-negativity had the lowest recurrence rate.	<0.001	Lack of standardization in ctDNA assays and definition of ctDNA positivity.	Meta-analysis of 28 studies
	Zhong [82]	2023	1251	ctDNA MRD detection can predict relapse with high specifity but suboptimal sensitivity, whether at specific time point or during interval surveillance. ctDNA surveillance significantly increases sensitivity with a slight decrease in specifity.	<0.05	Heterogenous group with SCLC and NSCLC patients.	Meta-analysis of 16 studies
	Pulla [106]	2024	461	ctDNA clearance prior to surgery is associated with pathological complete response benefit.	-	A need for confirmation in interventional studies.	Results confirmed with histopathological results
Perioperative treatment	Reck [89]	2024	283	ctDNA clearance prior to surgery identifies patients with improved event-free survival (EFS) with higher concordance than pCR status.	-	A need for confirmation in interventional studies.	Revolutionary data
	Wang [91]	2021	1017	Reduction in ctDNA levels during treatment is associated with longer OS and PFS in patients with advanced NSCLC receiving ICIs.	<0.001	Studies based on different drugs, high proportion of patients with *KRAS* and *TP53* mutations.	Meta-analysis of 10 studies
Treatment monitoring	Song [97]	2020	248	ctDNA clearance during the course of treatment is related to longer PFS and OS regardless of treatment type.	<0.001	Differences in timepoints of ctDNA evalution between studies.	Multicenter study comparing different treatment regimens
	Phan [100]	2022	4106	Detection of EGFR plasma mutations before treatment and after EGFR TKI initiation is negative prognostic factor for PFS and OS.	<0.001	Study considers the prognostic role of EGFR-plasma mutation as a single gene.	Meta-analysis of 35 studies

ctDNA—circulating tumor DNA; EGFR—epidermal growth factor receptor; EFS—event-free survival; ICIs—immune checkpoint inhibitors; MRD—minimal residual disease; NSCLC—non-small-cell lung cancer; OS—overall survival; pCR—pathological complete response; PFS—progression-free survival; SCLC—small-cell lung cancer; TKI—tyrosine kinase inhibitor; TP53—tumor protein p53.
